# Modeling Spatiotemporal Pattern of Depressive Symptoms Caused by COVID-19 Using Social Media Data Mining

**DOI:** 10.3390/ijerph17144988

**Published:** 2020-07-10

**Authors:** Diya Li, Harshita Chaudhary, Zhe Zhang

**Affiliations:** 1 Department of Geography, Texas A&M University, 3147 TAMU, College Station, TX 77843, USA; diya.li@tamu.edu; 2 Department of Computer Science and Engineering, Texas A&M University, 3112 TAMU, College Station, TX 77843, USA; harshita@tamu.edu

**Keywords:** COVID-19 pandemic, social media data mining, mental health, Basilisk algorithm, Patient Health Questionnaire (PHQ), Correlation Explanation (CorEx)

## Abstract

By 29 May 2020, the coronavirus disease (COVID-19) caused by SARS-CoV-2 had spread to 188 countries, infecting more than 5.9 million people, and causing 361,249 deaths. Governments issued travel restrictions, gatherings of institutions were cancelled, and citizens were ordered to socially distance themselves in an effort to limit the spread of the virus. Fear of being infected by the virus and panic over job losses and missed education opportunities have increased people’s stress levels. Psychological studies using traditional surveys are time-consuming and contain cognitive and sampling biases, and therefore cannot be used to build large datasets for a real-time depression analysis. In this article, we propose a CorExQ9 algorithm that integrates a Correlation Explanation (CorEx) learning algorithm and clinical Patient Health Questionnaire (PHQ) lexicon to detect COVID-19 related stress symptoms at a spatiotemporal scale in the United States. The proposed algorithm overcomes the common limitations of traditional topic detection models and minimizes the ambiguity that is caused by human interventions in social media data mining. The results show a strong correlation between stress symptoms and the number of increased COVID-19 cases for major U.S. cities such as Chicago, San Francisco, Seattle, New York, and Miami. The results also show that people’s risk perception is sensitive to the release of COVID-19 related public news and media messages. Between January and March, fear of infection and unpredictability of the virus caused widespread panic and people began stockpiling supplies, but later in April, concerns shifted as financial worries in western and eastern coastal areas of the U.S. left people uncertain of the long-term effects of COVID-19 on their lives.

## 1. Introduction

In December 2019, an outbreak of pneumonia caused by a novel coronavirus (COVID-19) occurred in Wuhan and spread rapidly throughout the globe [1]. The COVID-19 outbreak has forced people to change their regular routine lives and practice social distancing. Such a sudden change can drastically increase people’s stress level and lead to other mental health issues. The difficulties caused by the COVID-19 outbreak in different geographic regions can determine the cause and degree of stress in people, which corresponds to their risk of developing serious depression [2]. According to a poll [3], nearly half (45%) of adults in the United States reported that their mental health has been negatively impacted due to worry and stress over the virus. As the pandemic continues, it is likely that the mental health burden will increase as people’s sense of normalcy continues to be disrupted by social distancing, business and school closures, and shelter-in-place orders. The preexisting stress, constant unpredictability, and lack of resources lead to even greater isolation and financial distress.

Traditional mental health studies rely on information primarily collected through personal contact with a healthcare professional or through survey-based methods (e.g., via phone or online questionnaire). For instance, the Patient Health Questionnaire (PHQ) is a self-administered version of the Primary Care Evaluation of Mental Disorders (PRIME-MD) diagnostic instrument for common mental disorders [4]. However, these survey-based methods are time-consuming and suffer from cognitive and sampling biases, and therefore cannot be used to build large datasets for a real-time depression analysis [5]. Furthermore, understanding of spatial epidemic trends and geographic distribution patterns of COVID-19 provides timely information on people’s risk perception of epidemics. However, these important spatial and environmental leading factors are difficult to include in a survey-based method to model COVID-19 related mental stress.

Geographic Information System (GIS) and social media data mining have become essential tools with which to examine the spatial distribution of infectious diseases [6,7,8], and can be used to investigate the spatiotemporal pattern of mental stress caused by the pandemic. For instance, social media data (e.g., Twitter data) provide a unique opportunity to learn about the users’ moods, feelings, and behaviors that reflect their mental health as they experience daily struggles [8,9,10]. Many articles focused on using feature-based approaches to perform sentiment and emotional analysis using Twitter data [11,12,13,14]. For instance, Go and colleagues [11] investigated the usage of unigrams, bigrams, and their combination in training the classifiers for sentiment analysis of tweets. Various supervised classifiers were trained, including maximum entropy, naïve Bayes [15], and support vector machine (SVM) classifiers and their performance on the n-grams was compared. However, some methods previously used [11] have become outdated; for instance, they took emoticons into account for their sentiment index, but nowadays lots of Twitter users use emojis more frequently [16]. Barbosa and Feng [17] showed that n-grams are not useful in classifying tweets, as unused words in tweets can cause problems during classifier training. Pak and Paroubek [18] proposed the usage of microblogging features like hashtags, emoticons, re-tweets, and comments to train an SVM classifier and showed that it resulted in higher accuracy than training using n-grams. Several articles address the effect of using Part-Of-Search (POS) tag features in text classifiers [18,19]. Abadi and colleagues [19] investigated POS, lexicon, and microblogging features. The results showed that the most relevant features are those that combine prior polarity with the POS tags of the words. However, there have been mixed results reported on the usage of POS tags. Go and colleagues [11] showed that the POS tags caused reduced performance, although POS tags can be strong indicators of emotions in text and serve as a helpful feature in opinion or sentiment analysis [18]. Moreover, bootstrapping approaches, which rely on a seed list of opinion or emotion words to find other such words in a large corpus, are becoming more popular and have proven effective [20,21,22,23]. Mihalcea, Banea, and Wiebe [23] described two types of methods for bootstrapping the subjectivity lexicons into dictionary-based and corpus-based. Their research began with a small seed set of hand-picked subjective words, and with the help of an online dictionary produced a larger lexicon of potential candidate words. A similar bootstrapping model was effectively used to build a sentiment analysis system for extracting user-generated health review about drugs and medication [20]. However, all the aforementioned methods only detect the general emotion of tweets and lack the ability to model depression levels in detail.

Latent Dirichlet allocation (LDA) is one of the most commonly used unsupervised topical methods, where a topic is a distribution of co-occurring words [24]. However, the topics learned by LDA are not specific enough to correspond to depressive symptoms and human judgments [25]. The unsupervised method can work with unclassified text, but it often causes topics overlap [26]. Later, the LDA method was extended by using terms strongly related to PHQ-9 depression symptoms as seeds of the topical clusters and guided the model to aggregate semantically-related terms into the same cluster [27]. However, this approach only detects the presence, duration, and frequency of stress symptoms, ignoring the spatial context or environmental factors that are important in modeling the COVID-19 related mental stress. To identify PHQ related text and unrelated text, a sentiment analysis index generated by Python TextBlob was used [27], which only calculates the average polarity and subjectivity over each word in a given text using a constant dictionary [28,29]. Work based on the LDA probabilistic generative model was found to have limitations related to interpreting high dimensional human input factors which makes it difficult to generalize generative models without detailed and realistic assumptions for the data generation process [30,31,32].

In this article, we propose a CorExQ9 algorithm that integrates Correlation Explanation (CorEx) learning algorithm and clinical PHQ lexicon to detect COVID-19 related stress symptoms at a spatiotemporal scale in the United States. We aim to investigate people’s stress symptoms in different geographic regions caused by the development of the COVID-19 spread. Since Twitter data are high-dimensional human input data with diverse terms used to express emotions, we used the CorEx algorithm, a method intended to bypass the limitations of LDA implementation and minimize human intervention [33]. After that, we developed a fuzzy accuracy assessment model to visualize the uncertainty of the analytical results on the map.

The rest of the article is organized as follows: Section 2 introduces the material and methods used in the research work including the introduction of data collection and processing methods, Basilisk and machine learning classifier, and the proposed CorExQ9 algorithm. The results and discussion are presented in Section 3 and Section 4, respectively. Section 5 draws conclusions.

## 2. Material and Methods

### 2.1. Data Collection and Preprocessing

Twitter data used in this article were collected through the Twitter API from January to April 2020 for the continental United States. The collected data contained 80 million tweets (~70 GB), which posed significant computationally intensive challenges for the traditional GIS computing environment. To address this challenge, we used a Jupyter computing environment deployed on the Texas A&M High Performance Computer. We filtered the collected Twitter data using coronavirus related entities (e.g., hashtag, trends, and news). Then, we removed irrelevant information (e.g., non-English language Tweets, punctuation, missing data, messy code, URL, username, hashtags, numbers, and query terms) from the filtered tweets. Some adjustments and normalizations (e.g., uniform lower case, nonmaize vectorized tweets, standardize time sliced tweets) were also made in order to fulfill the common requirements of machine learning models. However, the stop words were removed later when applying the proposed algorithm to match the tweet phrase with lexicon. After that, the tweets were tokenized using the Natural Language Toolkit’s (NLTK) TweetTokenizer [33]. We also replaced repeated character sequences by using the length value of three for any sequences of length three or greater (3+), since most users often extend words or add redundant characters to express strong feelings. Tweets with an exact geospatial tag and timestamp were mapped to the corresponding county using reverse geocoding method [34,35]. Other tweets (e.g., without geotags but containing user-defined location information in the user’s profile) were geocoded to their corresponding county using a fuzzy set search method and city alias dataset [36]. We excluded tweets that did not have geotags nor user-defined location information. One of the key innovations in our research was to map the COVID-19 caused stress symptoms at a temporal scale. In this case, we set the temporal scale to biweekly starting from 26 January 2020, so the number of tweets collected in each county could be sufficient for accurate and reliable analysis.

### 2.2. Analytical Approach

#### 2.2.1. Bootstrapping the Initial Keywords

We used the Basilisk bootstrapping algorithm to find semantic lexicons that could be used to divide the tweets into two categories: stressed and non-stressed. The bootstrapping approach to semantic lexicon induction using semantic knowledge, also known as the Basilisk algorithm, was developed by Thelen and Riloff in 2002 [37]. This approach can extend to divide the tweets into multiple categories across different areas [22]. It employs a bootstrapping method to determine high-quality semantic lexicons of nouns. The algorithm takes a huge unannotated corpus from where it finds new related words and assigns them to the different semantic categories (e.g., stressed and non-stressed in our case). It is a form of categorization that is based on the seed words manually provided to the algorithm. These seed words are bootstrapped to identify new words that fall within the two categories.

Basilisk must be seeded with carefully selected terms for it to be effective. The two categories of seeds used for this task consisted of 20 words each (Table 1) [38]. The first category contained words describing stress and were used to bootstrap other words semantically related to stress or carrying a similar context. The second category contains words that describe non-stressed or a relaxing behavior. These two categories can be thought of as words that fall at the opposite ends of a stress level spectrum.

Before the bootstrapping process, the patterns were extracted on the unannotated corpus. This is used to extract all the noun phrases that were either the subject, direct object or prepositional phrase. The noun phrases were extracted from the corpus using the Stanford Dependency Parser [39]. It is a natural language parsing program used to find grammatical structure in sentences and can be used to find relationships or dependencies between nouns and the actions or words that form a group and go together. The dependency parser was run on all the sentences in the corpus and dependency relations were extracted for each word in the text (in the CoNLL-U format [40]).

For each tweet, the following dependency information was extracted. The CoNLL-U format of the extracted dependency pattern consists of the index, text, lemma, xpos, feats, governor, and dependency relations (Table 2). These extracted dependency relations were used to extract patterns that were used by the Basilisk algorithm to generate seeds. These extraction patterns were created for each dependency relation obtained in the previous step. The extraction patterns consisted of noun phrases and the dependency of them with other related words in the sentence. This acted as the input to the bootstrapping method.

After the input was generated, the next step was to generate the seeds using Basilisk. The seed words from the initial pattern pool enlarge with every bootstrapping step. The extraction patterns were scored using RlogF metric [41], which is commonly used for extraction pattern learning [41]. The score for each pattern was computed as: $RlogF\left(pattern_{\left(i\right)}\right)=\left(\frac{F_{i}}{N_{i}}\right)*\log_{2}F_{i}$, where $F_{i}$ represents the number of category members extracted by $pattern_{\left(i\right)}$ and $N_{i}$ is the total number of nouns extracted by $pattern_{i}$. This formula was used to score the patterns with a high precision or moderate precision but a high recall. The high scoring patterns were then placed in the pattern pool. After this process, all head nouns co-occurring with patterns in pattern pool were added to the candidate word pool. At the end of each bootstrapping cycle, the best candidates were added to the lexicon thus enlarging the lexicon set.

#### 2.2.2. Identify Stressed or Non-Stressed Tweets Using Words Obtained from Basilisk Algorithm

The process used related to Basilisk, as proposed by Thelen and Riloff, can be described using the algorithm shown on Table 3 (for notation description see Appendix A). This performs the categorization task of assigning nouns in an unannotated corpus to their corresponding semantic categories. Using the words generated by the Basilisk algorithm, we counted the total number of occurrences of any of the keywords in both categories. After the total count of stress and non-stress words in each tweet was obtained, we determined whether the tweet was in the category of stressed or non-stressed or neutral. This was done by finding the maximum of the stress and non-stress word counts in three conditions: (1) If there were more stress words than non-stress words, we annotated the tweet as expressing stress. (2) If the number of non-stress words is greater than the number of stress words, we annotated the tweet to express relaxed behavior. (3) If the count was zero for both stress and non-stress words, we did not annotate the data. Thus, tweets and their corresponding labels generated using this process were the initial training set, which was used to train a classifier to classify the other unannotated tweets.

#### 2.2.3. Generate Word Embeddings and Train the Classifier

The universal sentence encoder [42] was used to generate word embeddings. These text embeddings convert tweets into a numerical vector, encoding tweet texts into high dimensional vectors that are required to find semantic similarity and perform the classification task. It takes a variable length English text as input and outputs a 512-dimensional vector embedding. The encoder model was trained with a deep averaging network (DAN) encoder [15]. After the word embeddings were obtained for each stressed and non-stressed category tweet, a technique was used to make the two classes equalized. To do this, we selected the category with fewer samples and made the other category a similar size by removing samples. This ensured that the training process was not biased towards a particular class.

Before training the classifier, the data were split into training and validation sets. The data were randomly shuffled and put into the two datasets, with 80% used as the training dataset. To obtain the best performance, multiple classifiers were used, and performance was compared using accuracy metrics. The classifiers used in the training process were SVM [42], logistic regression [43], naïve Bayes classifier [44], and a simple neural network.

SVM handles nonlinear input spaces and separates data points using a hyperplane using the largest amount of margin. As a discriminative classifier, SVM found an optimal hyperplane for our data, which helped with classifying new unannotated data points. We used different kernels to train the SVM. The hyperparameters were tuned and the optimal value of regularization and gamma were also recorded. The logistic regression classification algorithm can be used to predict the probability of a categorical dependent variable. The dependent variable is a binary variable that contains data coded as 1 (stressed) or 0 (non-stressed). The logistic regression model predicts P(Y=1) as a function of X. Prior to training, it shuffles the data. It uses a logistic function to estimate probabilities to calculate the relationship between independent variable(s) and the categorical dependent variable [45]. Naïve Bayes is another probabilistic classifier which makes classifications using the Bayes rule. This classifier is simple and effective for text classification. A simple neural network consisting of three dense layers were used to train our datasets. The loss function and optimizer used in the training is binary cross entropy and RMSProp, respectively. Training was done for 40 epochs with a batch size of 512. Table 4 illustrates the performance evaluation of these classifiers.

#### 2.2.4. Generate Labels Using the Trained Classifier

After the model was trained, the model was run on the unannotated tweets to label them. To label the sentence embeddings for the tweets, the same procedure was used as for the training set. The universal sentence encoder extracts 512 features and created vectors that were used to classify the tweets based on the model. The SVM classifier with linear kernel was used to predict the probabilities of the tweets because it had the best trained models (see Table 4). Here, a threshold of 0.75 was set to determine if the tweet belonged to a particular category or not. If the probability of the tweet was above 0.7 for that category, the tweet was classified with the corresponding label.

The tweets and labels generated using the above process were then used to train another classifier to generate the final model for classification of the entire unannotated corpus. Here, a logistic regression model was used to train tweets and their corresponding labels generated using the above process to ensure that the model was robust and was not overfitted on the initial set of tweets that were filtered out using the Basilisk generated keywords. The trained model had an accuracy of 90.2% on the validation data.

#### 2.2.5. CorExQ9 Algorithm

In this article, we propose a novel CorExQ9 algorithm to detect spatiotemporal patterns of COVID-19 related stress. Table 5 illustrates the general structure of the CorExQ9 algorithm. The input of the algorithm was the stressed-related tweets derived by using the trained models (see Section 2.2.3 and Section 2.2.4) to all the processed COVID-19 related tweets. We assessed the level of stress expressed in COVID-19 related tweets by integrating a lexicon-based method derived from established clinical assessment questionnaire PHQ-9 [46]. Table 6 illustrates the PHQ-9 lexicon examples and their corresponding mental stress symptoms.

The PHQ-9 lexicon contains about 1700 clinical words, which is difficult to understand and match with the spoken language that is often used on Twitter. Therefore, we used the following methods to transform PHQ-9 lexicon to human understandable language by appending matched tweets to their best match PHQ-9 categories. In the first step, each tweet was placed into a set of phrase sets using Natural Language Processing toolkit spaCy [44] (see Table 4, procedure 1). After that, the tweets and PHQ-9 lexicon were vectorized using Global Vectors for Word Representation (GloVe), Wikipedia, and Gigaword 5 model (with 300 dimensional word vectors and four million unique tokens) [45]. GloVe provides a quantitative way to distinguish the nuance difference of two words (e.g., happy or unhappy), which is useful to match phrases set with the PHQ-9 lexicon. Those pre-trained vectors were loaded to Gensim [47] to perform average vector and cosine distance calculation (see Equations (1) and (2)). We appended all phrases that have the similarity rate higher than 0.8 to their corresponding PHQ-9 lexicon (see Table 4, procedures 3–5).

Given any words in a phrase, the average vector was calculated using the sum of the vectors divided by the number of words in a phrase:

(1) $$V=average\;vector=\frac{sum\left(vectors\right)}{number\;of\;words\;in\;a\;phrase}$$

Given any two average vectors $V_{A}$ and $V_{B}$ of two phrases, the cosine similarity, cos θ, is represented by

(2) $$Similarity=\cos\theta =\frac{\sum_{i=1}^{n}V_{A}{}_{i}V_{B}{}_{i}}{\sqrt{\sum_{i=1}^{n}V_{A}{}_{i}^{2}}\sqrt{\sum_{i=1}^{n}V_{B}{}_{i}^{2}}}$$

Next, a sparse matrix (e.g., a vocabulary dense matrix) for stressed corpus was calculated by transforming those tokenized and vectorized tweets using frequency inverse document frequency (TFIDF). The mathematical formula of TFIDF is illustrated below:

(3) tfidf(t,d,D)=tf(t,d)∗idf(t,D),

where t denotes the terms; d denotes each document; and D denotes the collection of documents. The first part of the formula tf(t,d) calculates the number of times each word in COVID-19 corpus appeared in each document. The second part of idf(t,D) is made up with a numerator $D=d_{1},d_{2},\ldots d_{n}$ and a denominator |{d∈D:t∈d}|. The numerator infers the document space, which is all documents in our COVID-19 stress corpus. The denominator implies the total number of times in which term t appeas in all of our documents d. The idf(t,D) can be represented by

(4) $$idf\left(t,D\right)=\log\frac{\left|D\right|}{1+\left|\left\{d\in D:t\in d\right\}\right|}$$

We utilized Scikit-Learn TfidfVectorizer to transform preprocessed tweets to a sparse matrix [48] (see Table 4, procedure 6). After that, the sparse matrix and lexicon are used by the anchored CorEx model to perform anchored topic modeling [32]. The total correlation TC [49] (for notation description see Appendix A) of each topic is calculated by anchoring the CorEx model with the document sparse matrix. The total correlation in our PHQ-9 lexicon detection can be expressed using Kullback–Leibler divergence as below [50].

(5) $$TC\left(X_{G}\right)=D_{KL}\left(p\left(x_{G}\right)||\prod_{i\epsilon G}p\left(x_{i}\right)\right),$$

where $p(x_{G})$ represents the probability distribution and $TC\left(X_{G}\right)$ is non-negative or zero factorizes of $p(x_{G})$ (see Appendix A for more detail). In the context of PHQ-9 detection, $X_{G}$ represents the group of word types among the COVID-19 corpus. Note that each vector in the TFIDF matrix is based on the distance between two probability distributions, which is expressed as cross-entropy Entropy(X) [51,52]. When introducing a random variable Y, the TC can explain the correlation reduction in X, which is a measure of the redundant information that the word types X carry about topic Y [30]. The total correlation can be represented by:

(6) $$TC\left(X_{G};Y\right)=TC\left(X_{G}\right)-TC\left(X_{G}|Y\right)=\sum_{i\epsilon G}I\left(X_{i}:Y\right)-I\left(X_{G}:Y\right),$$

where I(X:Y)=Entropy(X)+Entropy(Y)−Entropy(X, Y) (for notation description see Appendix A).

Thus, the algorithm starts with randomly initialized variables $\alpha_{i,j}$ and $p(y_{i}|x_{i})$, where $\alpha_{i,j}$ are indicator variables of TC that are assigned to 1 if the topic is detected and $p\left(x_{i}\right)$ represents the approximate empirical distribution (see Table 4, procedure 7). Then, the correlation explanation updates both variables iteratively until the result achieves convergence. In each iteration, the estimate marginals $p(y_{j}|x_{i})=\sum_{\bar{x}}\;p(y_{i}|\bar{x})p\left(\bar{x}\right)\delta_{{\bar{x}}_{i}}$ and mutual information TC are calculated (notation description see Appendix A). Next, the update for $a_{i,j}^{t}$ in each t step is calculated by

(7) $$a_{i,j}^{t}=\exp\left(\lambda^{t}\left(I\left(X_{i}:Y_{j}\right)-\underset{\bar{j}}{max}I\left(X_{i}:Y_{\bar{j}}\right)\right)\right),$$

where λ conduct a smooth optimization of the soft-max function [53,54]. Finally, the soft labeling of any x (for notation description see Appendix A) can be computed by

(8) $$p\left(y_{j}|x\right)=\frac{1}{Z_{j}\left(x\right)}p\left(y_{j}\right)\overset{n}{\underset{i=1}{\prod }}{\left(\frac{p\left(y_{j}|x_{i}\right)}{p\left(y_{j}\right)}\right)}^{\alpha_{i,j}}$$

After the soft-max function α converges to the true solution at a particular step $\alpha^{k}$ in the limit λ→∞, the mutual information terms can be ranked by the informative order in each factor. To perform semi-supervised anchoring strategies, Gallagher and Reing proposed the combination with bottleneck function and total correlation [32]. The bottleneck function can be represented by:

(9) $$B=\underset{p\left(y|x\right)}{\max}\beta I\left(Z:Y\right)-I\left(X:Y\right)$$

The connection with CorEx and anchor words can be described by comparing Equation (6) with Equation (9). The same term I(X:Y) in two equations represents the latent factor and the variable Z corresponds to $X_{i}$. It is worth noting that Z is typically labeled in a supervised learning task [54] and β is a constant parameter to constrain supervising strength so that α=β can imply a word type $X_{i}$ correlated with topic $Y_{j}$. In this case, Z was represented by each variable generated by the enriched PHQ-9 lexicon. To seed lexicon to detect topics, we can simply anchor the word type $X_{i}$ to topic $Y_{j}$, by constraining the β (see Table 5, procedures 8–11).

#### 2.2.6. Define the PHQ Category and Uncertainty Analysis

The symptoms of COVID-19 related stress were visualized at the county level biweekly from 26 January. Here, we used the fuzzy accuracy assessment method to evaluate the uncertainty of final PHQ stress level for each county [55,56].

We summarized the implementation of fuzzy accuracy assessment for a thematic map as presented by Gopal and Woodcock to explain our model evaluation for the PHQ map [55]. Let X be a finite universe of discourse, which is the set of county polygons in the study area. Let ζ denote the finite set of attribute membership function (MF) topics categories to the d in X; and let m be the number of categories |ζ|=m, (e.g., nine PHQ categories). For each x ϵ X, we define χ(x) as the MF classes assigned to x. The set:

(10) M={x,χ(x)| x ϵ X},

defines the data. The subset S⊂X of n data is used. A fuzzy set is associated with each class C ϵ ζ where $\mu_{c}\left(x\right)$ is the characteristic of MF of C. The fuzzy set can be represented as:

(11) $$A_{c}=\{(x,\;\mu_{c}\left(x\right)|x\;\epsilon \;S\}$$

To implement a decision-making system for fuzzy accuracy, the model uses a Boolean function σ that returns results of 0 or 1 based on whether x belongs to the class C with respect to the matrix A. That is, σ(x, C) = 1 if x “belongs” C, and σ(x, C)=0 if x does not “belong” to C. Then σ(x, C) is 1 if the numeric scale of the MF for x in category $C\left(\mu_{c}\left(x\right)\right)$ is maximum among all map categories $\mu_{c^{\prime }}\left(x\right)$, and we set the Boolean function σ as MAX follows:

(12) $$MAX\left(x,\;C\right)=\{\begin{array}{c} 1\;\left(if\;\mu_{c}\left(x\right)\geq \;\mu_{c^{\prime }}\left(x\right)for\;all\;C^{\prime }\epsilon \;\zeta \right)\;\\ 0\;\left(otherwise\right) \end{array}$$

According to the fuzzy set accuracy assessment, the final PHQ value for each county was selected based on the MAX function, meaning each county was colored based on the majority tweet PHQ value derived from the proposed CorExQ9 algorithm. Since the accuracy assessment was based on a comparison of the PHQ label assigned to each county with the evaluation given by the expert (e.g., in each county, the majority tweet PHQ label). The rating system can thus be expressed as linguistic variables that describe the uncertainty associated with the evaluation of the class label. Here, the linguistic variables are described below:

1. Score 1: Understandable: the answer is understandable but may contain high levels of uncertainty;
2. Score 2: Reasonable: maybe not the best possible answer but acceptable;
3. Score 3: Good: would be happy to find this answer given on the map;
4. Score 4: Absolutely right: no doubt about the match. It is a perfect prediction.

**Figure 1:**
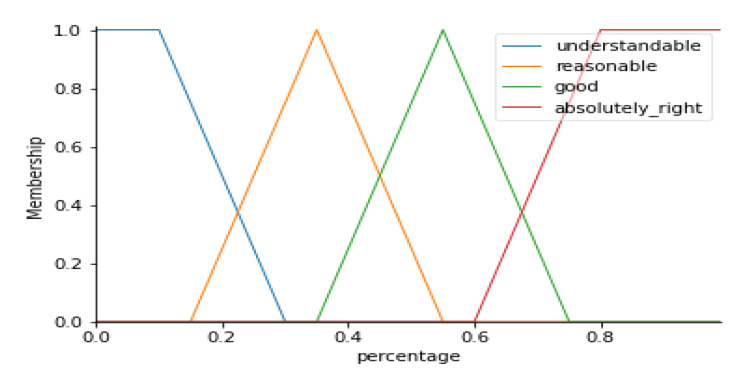
Fuzzy membership functions of uncertainty evaluation of assigned PHQ category.

Figure 1 illustrates the fuzzy MF created for the fuzzy accuracy assessment analysis. The x-axis represents the percentage of the tweets that belong to the assigned final PHQ category. The y-axis represents the value of the degree of the membership function corresponding to the linguistic score. For instance, if a county was assigned to a PHQ category 3, and 80% (e.g., x = 0.8 in Figure 1) of the tweets within this county polygon were labeled as PHQ-3 using the CorExQ9 algorithm, the corresponding MF should be absolutely right with membership value equal to 1. The accuracy assessment score was further visualized on the PHQ stress map to show the spatial uncertainty of the analysis results.

### 2.3. Baseline Evaluation

Since CorExQ9 represents topic and potential symptoms as a lexicon-based topic modeling, traditional measures such as regression correlation and log-likelihood are unnecessary for the semantic topics. Therefore, to evaluate the baseline performance of the CorExQ9 model, we first involved the semantic topic quality coherence measure methods with other common topic models. We compared CorExQ9 with LDA and non-negative matrix factorization (NMF) [57,58]. In addition, we used Frobenius normalized NMF (NMF-F) and generalized Kullback–Leibler divergence NMF (NMF-LK) for a closer comparison with traditional topic modeling. All models were trained with a randomly selected COVID-19 Twitter dataset. The topics generated by those models were scored by topic coherence measures to identify the degree of semantic similarity between high-scoring words in the topic. A common coherence measure is UMass which calculates and scores the word co-occurrence in all documents [59]:

(13) $$score_{UMass}\left(w_{i},\;w_{j}\right)=log\frac{D\left(w_{i},\;w_{j}\right)+c}{D\left(w_{i}\right)}$$

where D$\left(w_{i},\;w_{j}\right)$ represents the number of documents containing both $w_{i}$ and $w_{j}$ words and $D\left(w_{i}\right)$ counts the ones containing $w_{i}$, and c represents a smoothing factor. The intrinsic UMass [59] coherence measure calculates these probabilities over the same training corpus.

Additionally, the extrinsic UCI measure [58] introduced by David Newman uses a pairwise score function, which is based on pointwise mutual information (PMI). It can be represented by:

(14) $$score_{UCI}\left(w_{i},\;w_{j}\right)=PMI\left(w_{i},\;w_{j}\right)=log\frac{p\left(w_{i},\;w_{j}\right)}{p\left(w_{i}\right)p\left(w_{j}\right)}$$

where $p\left(w_{i}\right)$ represents the probability of seeing $w_{i}$ in a random document, and $p\left(w_{i},\;w_{j}\right)$ is the probability of seeing both $w_{i}$ and $w_{j}$ co-occurring in a random document. Those probabilities are empirically estimated from an external dataset such as Wikipedia. The higher the topic coherence measure score, the higher the quality of the topics. In our baseline evaluation, we calculated the coherence scores by setting the range of topic numbers from 10 to 30. The abnormal and low-quality topics were cleared and the average coherence scores (Table 7) were calculated by the sum of all coherence scores divided by the number of topics. On average, the CorExQ9 algorithm has a better UMass score than LDA and NMF. Even though the UCI score was slightly lower than two types of NMF algorithms, we can take the external estimation dataset as an uncertainty factor of this coherence model because the result of the comparison was still meaningfully coherent and it has the competitive functionality of the semi-supervised feature, which exceeded the usable range of NMF.

## 3. Results

### 3.1. Overall Experimental Procedures 

**Figure 2:**
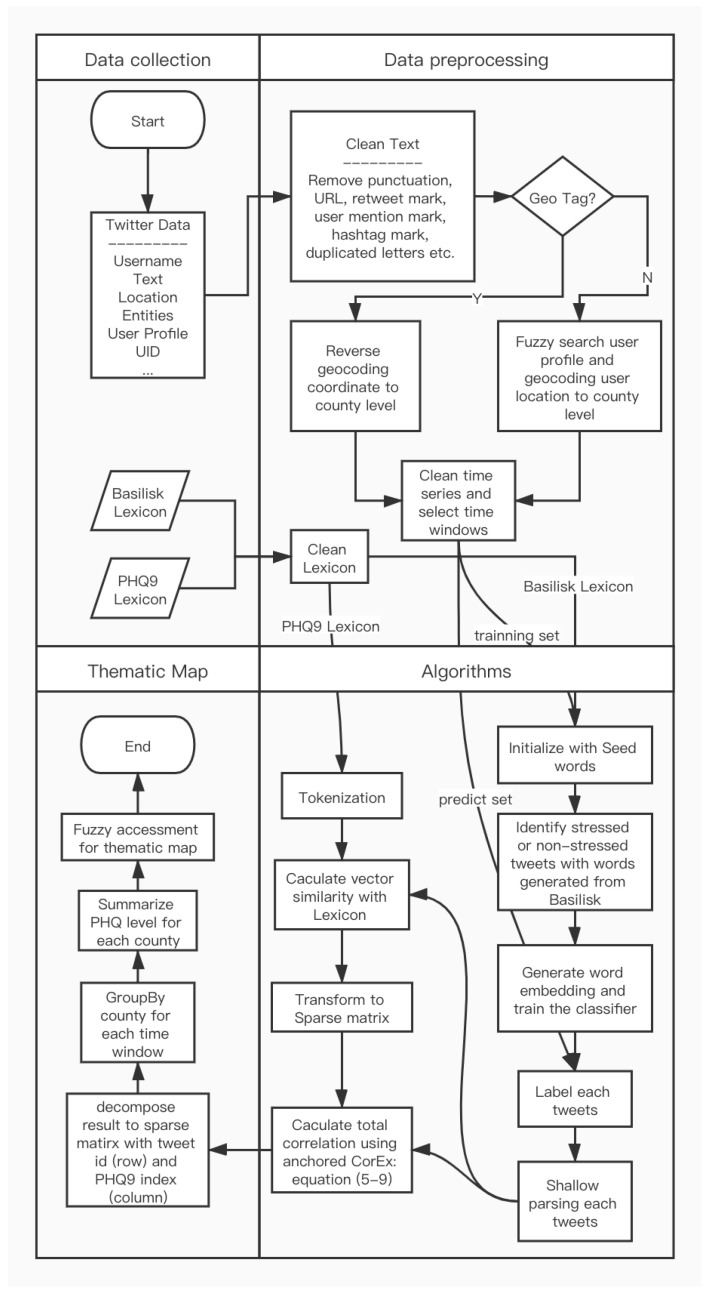
Process undertaken to generate spatiotemporal stress symptom maps and topics.

In our research, the methods described above were combined to generate the final thematic map. To summarize processes for each detailed procedure, the workflow for the research is shown in Figure 2. First, starting from data collection, we prepared a Twitter dataset, Basilisk lexicon, and PHQ-9 lexicon. Then, we cleaned each tweet and extracted its location information using the method mentioned in Section 2.1. To engage time series analysis, the whole Twitter dataset was formatted and sorted by Unix Timestamp before being sliced into two-week intervals. Third, two lexicons were separately assigned to CorExQ9 and Basilisk algorithm (mentioned in Section 2.2) with the prepared Twitter dataset. In the end, we decomposed the result generated by anchored CorEx model into spare matrix in order to group by all tweets in county level for visualization. Note that each row of the results from the CorEx algorithm represents the correlations index within an individual tweet explained by nine PHQ levels so that we can reverse convert the result to its original tweets. The selected top symptoms and topics are present in Table 8.

### 3.2. Fuzzy Accuracy Assessment Results

**Figure 3:**
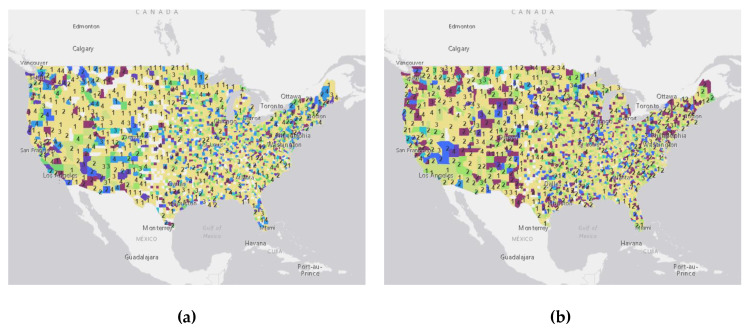

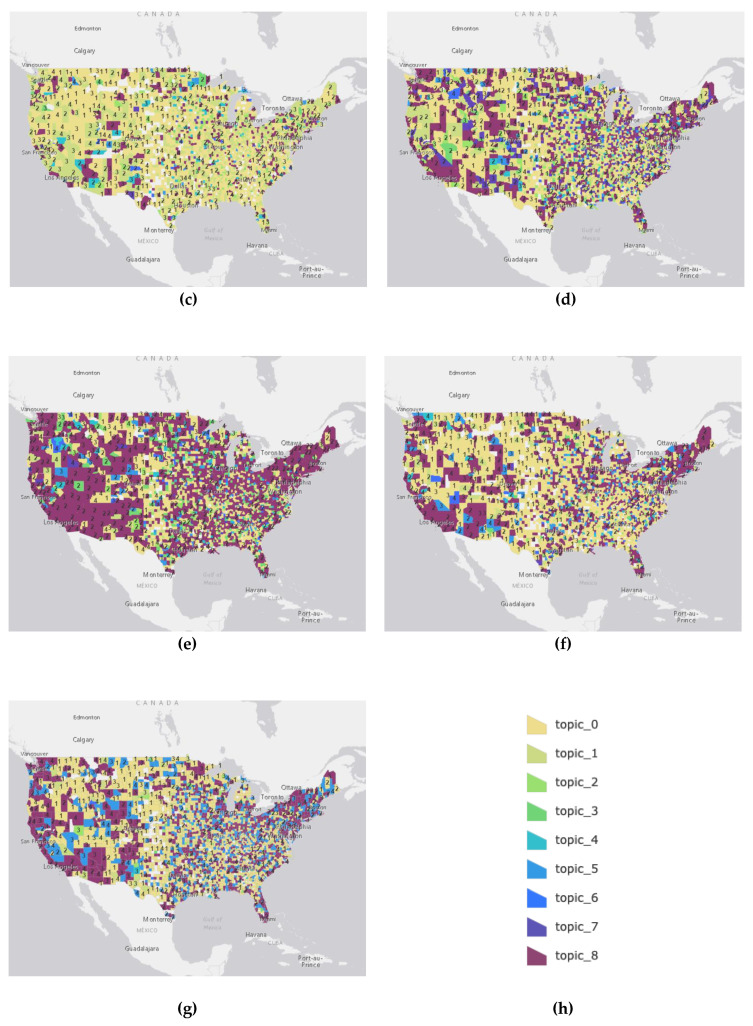
Spatiotemporal pattern with fuzzy accuracy assessment for stress symptom analysis result generated by CorExQ9. (**a**) from 01.26.2020 to 02.09.2020; (**b**) from 02.09.2020 to 02.23.2020; (**c**) from 02.23.2020 to 03.08.2020; (**d**) from 03.08.2020 to 03.22.2020; (**e**) from 03.22.2020 to 04.05.2020; (**f**) from 04.05.2020 to 04.19.2020; (**g**) from 04.19.2020 to 05.03.2020; (**h**) The legend of for (a)–(g).

The fuzzy accuracy assessment results of the study are illustrated in Figure 3. On each map, the individual county is colored according to the assigned PHQ index using the proposed algorithm and fuzzy assessment accuracy assessment method. The numbers on the map represent the spatial uncertainty indices derived from the fuzzy accuracy assessment. Each number represents the assessment score calculated from Section 2.2.6. For most of the hot spots areas in Figure 3, the values are greater than two, which indicates middle to high accuracy results have been reached for those regions. Higher scores for an area indicate a larger percentage of the topics being present in this area at specific time region.

### 3.3. Spatiotemporal Patterns and Detected Topics

The results also present the spatiotemporal patterns from January to April (shown in Figure 3a–g. Table 8 shows the detected stress symptoms and topics generated from CorExQ9. Each map represents the spatial distribution of stress symptoms over a biweekly period. It indicates that most of the regions have low to medium PHQ values (topic 0–3) during January and February, since information about the U.S. COVID-19 outbreak was not publicly available in the U.S. during that time. Most counties that have a low PHQ level contain general COVID-19 related topics that are tied to the cases in Asia and general symptoms of COVID-19 (e.g., “Wenliang Li” (a Chinese doctor) [60], “South Korea confirms”, “coughing”, “sneezing”). From the end of January, a few hotspots appear in some major U.S. cities such as San Francisco, Denver, Los Angeles, and Seattle with topics related to “mistakenly released”, “vaccine”, “pandemic bus”, and “China death” (see Table 8, Figure 3b,c). For instance, the keyword “mistakenly released” reflects news story in February about the first U.S. evacuee from China known to be infected with the coronavirus being mistakenly released from a San Diego hospital and returned to quarantine [61]. People who living in California reacted strongly to this news (Figure 3d).

**Figure 4:**
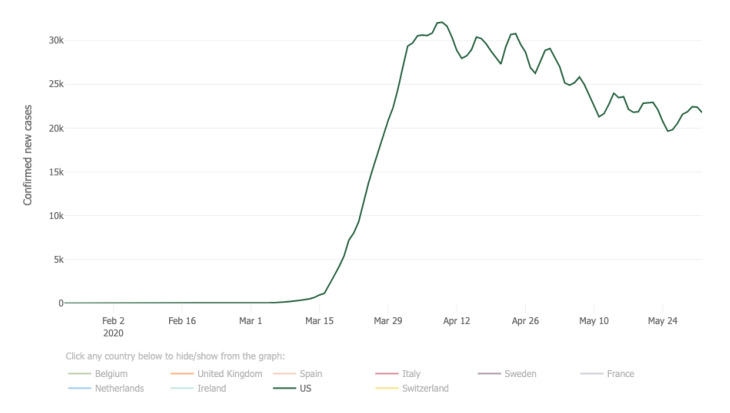
The number of daily confirmed new cases in the United State (five-day moving average) [63,64].

Later, on 8 March (Figure 3c,d), the PHQ level started to increase rapidly due to the COVID-19 test stations available, increased number of COVID-19 death cases, and a shelter-in-place order in many states (see Table 8, March). An interesting pattern was found that the number of counties with a high PHQ value kept growing until 5 April and started to decrease after the second week of April [62]. Figure 4 illustrates the number of increased cases in the U.S. from January to May 2020. Results show that the PHQ stress level in our results matches well with the number of increased cases illustrated in the Johns Hopkins Coronavirus Resource Centers’ statistical analysis results [61]. This means the number of new cases reduced due to the social distancing practice, and at the same time, the level of people’s major concerns in many geographic regions reduced as well.

Our results also show a meaningful explanation of the spatial pattern caused by people’s risk perception to various media messages and news during the pandemic. In March 2020, people in the United States had mild concerns about the UK prime minister Boris Johnson’s talk of “Herd Immunity” [65] and social distancing (see Table 8, PHQ0, March). On the other hand, the major stress came from topics such as cases of deaths (e.g., in Washington State), lack of food and COVID-19 protection equipment (e.g., panic buy), and the increasing number of confirmed and death cases in the United States. Figure 3d,e shows that most of the hotspots were located in Washington, California, and New York, and Florida matched with to the March COVID-19 increased cases map (see [61]. In April, keywords such as “death camps”, “living expenses”, “white house”, and “economy shrinks” (see Table 8) appeared most often in the high PHQ value categories, which indicated that people’s major concerns shifted to financial worries due to businesses shutting down and the economic depression [66].

## 4. Discussion

Our study was conducted to perform a spatiotemporal stress analysis of Twitter users during COVID-19 pandemic by the CorExQ9 algorithm. According to the model evaluation results, the proposed CorExQ9 had the best baseline performance among other similar algorithms such as LDA, NMF-LK, and NMF-F models. In addition to the CorExQ9 algorithm, we applied a fuzzy accuracy assessment method to the CorExQ9 analysis results to visualize the spatial uncertainty of the analysis results. This enables expert knowledge (e.g., PHQ rating of tweets) to be integrated in the social media data mining process. The result of our observed pattern reasonably matched the relevant events and epidemic trends.

Ideally, the analytic result of our collected Twitter dataset is expected to support the research of mental health for the entire U.S. population as a sample case. In our cleaned Twitter dataset, those tweets were posted by 1,410,651 users, which represent over 0.4% of the U.S. population. However, a previous investigation found that the 22% of American adults who use Twitter are not uniformly distributed across age [66,67]. Another study found that Twitter users are getting younger [68], but the actual age, gender, and race of Twitter users from those investigations have been controversial [55]. To generalize the psychology health analysis to the whole U.S. population, further work related to the user demographic is required to reduce the influence of the sample bias.

The thematic maps we created for PHQ topics distribution were assessed based on fuzzy sets. The purpose of this commonly used method for categorical maps is to allow explicit accounts for the possible ambiguity regarding the appropriate map label [55,69,70,71,72]. A wide variety of statistical techniques have been proposed for the accuracy assessment of thematic maps [73]. In the future, we can use the standard deviation approach to estimate the quantity derived from the distribution of the tweets as a count on specific category if the assessment is focused on how the number of labeled PHQ tweets were distributed in each category. Even though our datasets were preprocessed and selected with entities on COVID-19 related topic, some of the tweets might be outside of the topic or are influenced by other objective factors. Our future focus of uncertainty assessment of the thematic maps could be to extend to spatial uncertainty [74], temporal uncertainty [75] semantic uncertainty [76], etc. Our assessment task can be considered a criterion referenced task that can focus on a selected PHQ level and can represent the majority level in any location. The fuzzy area estimation methods were extended based on previous research [72]. Category assessment based on fuzzy sets can estimate the accuracy of classes as a function of levels of class membership [77].

Here, we used biweekly data as a temporal scale for the analysis. Our research group continues collecting Twitter data for this project, so analysis could be applied to more fine-grained temporal scales in the future. Since COIVD-19 is a global pandemic, this project could be extended to a global scale to compare the results across different countries. In the future, the model could be applied to other cases to detect the related stress symptoms and provide real-time spatial decision support for addressing the problem. An end-to-end spatiotemporal analysis system could be built if all of the modules were integrated; this would increase the efficiency of determining the potential symptoms and causes of public mental health problems.

## 5. Conclusions

In this article, we proposed the CorExQ9 algorithm to analyze the COVID-19 related stress symptoms at a spatiotemporal scale. The CorEx algorithm combined with clinical stress measure index (PHQ-9) helped to minimize human interventions and human language ambiguity in social media data mining for stress detection and provided accurate stress symptom measures of Twitter users related to the COVID-19 pandemic. There was a strong correlation between stress symptoms and the number of increased new COVID-19 cases for some major U.S. cities such as Chicago, San Francisco, Seattle, New York, and Miami. People’s risk perceptions were sensitive to the release of COVID-19 related public news and media messages. Many frequently appearing keywords in the high PHQ value categories represent the popular media and news publications at that time. Before March, most regions had mild stress symptoms due to the low number of reported cases caused by the unavailability of test stations, creating a false sense of security among the public in the United States. The number of cases increased suddenly in March due to governmental confirmation of the seriousness of the pandemic in the United States and shelter-in-place orders in many states. From January to March, a major concern for people was being infected by the disease and there was panic-buying behavior, but this shifted to financial distress later in April along coastal eastern and western United States.

Our main contributions are as follows: First, we introduced a specialized stress tweets classifier, which narrows down the theoretical algorithms to practical usage on the public health area and demonstrates more effectiveness than traditional sentiment index classifiers. Second, we framed CorExPQ9 as a topic detection model in our research. We explored the latent connection between the social media activity and PHQ-9 depression symptoms and topics in United States. Finally, as a supplement methodology for the existing questionnaire-driven mental health research, our integrated system was used to glean depression topics in an unobtrusive way.

The proposed algorithm provides an innovative way to analyze social media data to measure stress symptoms under COVID-19 pandemic at a spatiotemporal scale. By doing this, we were able to observe spatiotemporal patterns of stress symptoms and answer the questions of what the major concerns related to the pandemic in different geographic regions at different time scales were. In the future, this model could be applied to other cases to detect related stress symptoms and provide real-time spatial decision support for addressing arising issues.

## Figures and Tables

**Table 1:** Illustration of seed words for the Basilisk algorithm [37].

**Initial Stressed Seed Words:**						
addiction	boredom	dissatisfaction	grief	insecure	fear	stress
tense	burnout	meditation	guilt	irritable	panic	alcoholism
anger	conflict	embarrassment	headache	irritated	pressure	tension
anxiety	criticism	communication	tired	loneliness	problem	impatience
backaches	deadline	frustration	impatient	nervous	sadness	worry
**Initial Non-Stressed Seed Words:**						
chill	satisfaction	self-confidence	cure	perfection	heart	prevention
enjoy	happiness	self-improvement	distress	overwork	right	self-talk
love	productivity	empowerment	wedding	perfectionism	change	tension
relax	perfection	self-image	marriage	self-help	family	tired
relaxation	well-being	commitment	relax	control	joy	empower

**Table 2:** Description of dependency information.

Information in Text	Description
Index	Index of the word in the sentence
Text	Text of the word at the particular index
Lemma	Lemmatized value of the word
Xpos	Treebank-specific part-of-speech of the word. Example: “NNP”
Feats	Morphological features of the word. Example: “Gender = Ferm”
Governor	The index of governor of the word, which is 0 for root
Dependency relation	Dependency relation of the word with the governor word which is root if governor = 0. Example: “nmod”

**Table 3:** Illustration of the Basilisk algorithm [41].

**Input:** *Extraction Patterns in the Unannotated Corpus and their Extractions, Seed Lists* **Output:** *Updated List of Seeds*
Procedure: lexicon={seed words} for i:=0 1. Score all extraction patterns with RlogF 2. pattern pool = top ranked 20+i patterns 3. candidate word pool = extractions of patterns in pattern pool 4. Score candidate words in candidate word pool 5. Add top five candidate words to lexicon 6. i:=i+1 7. Go to Step 1.

**Table 4:** Performance evaluation of classifiers.

Model	Validation Accuracy
Support Vector Machine (SVM) (Radial basis function kernel)	0.8218
SVM (Linear kernel)	0.8698
Logistic Regression	0.8620
Naïve Bayes	0.8076
Simple Neural Network	0.8690

**Table 5:** Illustration of Correlation Explanation (CorEx)Q9 algorithm.

**Input:***phq_lexicon, *Stressed* Tweets (geotagged)* **Output:***topic sparse matrix S where row: tweet_id_ and columns: PHQ Stress Level Index* (1 *to* 9)
Procedure: 1. Shallow parsing each tweet into tweet_pharse using spaCy 2. For each word_set in phq_lexicon do 3. Calculate average vector of word_set and tweet_pharse using GloVe 4. Match word_set with tweet_pharse set using cosine similarity measure 5. Append each matched tweet_pharse to word_set 6. Calculate Tf-Idf vector for all the tweets and transform the calculated value to a sparse matrix X 7. Iteratively run CorEx function with initial random variables $V_{random}$ 8. Estimate marginals; calculate total correlation; update $V_{random}$ 9. For each word_set in phq_lexicon 10. Compare $V_{random}$ and word_set with bottleneck function 11. Until convergence

**Table 6:** Patient Health Questionnaire (PHQ)-9 lexicon description and examples.

PHQ-9 Category	Description	Lexicon Examples
PHQ1	Little interest or pleasure in doing things	Acedia, anhedonia, bored, boring, ca not be bothered
PHQ2	Feeling down, depressed	Abject, affliction, agony, all torn up, bad day
PHQ3	Trouble falling or staying asleep	Active at night, all nightery, awake, bad sleep
PHQ4	Feeling tired or having little energy	Bushed, debilitate, did nothing, dog tired
PHQ5	Poor appetite or overeating	Abdominals, anorectic, anorexia, as big as a mountain
PHQ6	Feeling bad about yourself	I am a burden, abhorrence, forgotten, give up
PHQ7	Trouble concentrating on things	Absent minded, absorbed, abstracted, addled
PHQ8	Moving or speaking so slowly that other people could have noticed	Adagio, agitated, angry, annoyed, disconcert, furious
PHQ9	Thoughts that you would be better off dead	Belt down, benumb, better be dead, blade, bleed

**Table 7:** Average coherence measure score.

Model	Average UMass	Average UCI
CorExQ9	–3.77	–2.61
LDA	–4.22	–2.76
NMF-LK	–3.97	–2.58
NMF-F	–4.03	–2.36

Abbreviations: UCI = The UCI measure was first introduced by researches in University of California, Irvine; UMass = Umass measure was first introduced by researches in University of Massachusets. Related papers using these measures are just using UCI and Umass directly; LDA = latent Dirichlet allocation; NMF-LK = Kullback-Leibler divergence non-negative matrix factorization; NMF-F = Frobenius normalized NMF.

**Table 8:** Illustration of detected stress symptoms based on PHQ-9 category.

PHQ-9 Category and Description	Top Symptoms and Topics
PHQ0: Little interest or pleasure in doing things	**Feb.:** Chinese journalist, koalas, snakes, Melinda gates, South Korea, World Health Organization (WHO) declared outbreak, send hell, airways suspended, etc. **Mar.**: Prime Minister Boris, Dr. Anthony Fauci, moved intensively, attending mega rally, Tom Hanks, Rita Wilson, etc. **Apr.:** stay home, bored at home, masks arrived pos, sign petition UK change, uninformed act, etc.
PHQ1: Feeling down, depressed	**Feb.:** Wenliang Li, whistleblower, South Korea confirms, suffering eye darkness, China breaking, global health emergency, Nancy Messonnier, grave situation, etc. **Mar.:** abject, despair, Kelly Loeffler, Jim, stock, Richard Burr, feeling sorry, Gavin Newsom, cynical, nazi paedophile, destroyed, etc. **Apr.:** social isolation, ha island, suffering, bus driver, coverings, cloth face, etc.
PHQ2: Trouble falling or staying asleep	**Feb.:** sneezing, coughing, avoid nonessential travel, diamond princess cruise, San Lazaro hospital, RepRooney, Dean Koontz, gun, arranging flight, etc. **Mar.:** calls grow quarantine, secret meetings, donates quarterly, task force, sleepy, cutting pandemic, nitrogen dioxide, aquarium closed, Elba tested, etc. **Apr.:** workers, healthcare, basic income, Bronx zoo, tiger, keep awaking, coughing, concealed, etc.
PHQ3: Feeling tired or having little energy	**Feb.:** test positive, tired dropping flies, horror, clinical features patients, national health commission, governors, flown CDC advice, weakness, etc. **Mar.:** blocking bill limits, drugmakers, Elizabeth fault, CPAC attendee tested, overruled health, collapses, front lines, practicing social distancing, etc. **Apr.:** exhausted, Boris Johnson admitted hospital, Brooke Baldwin, etc.
PHQ4: Poor appetite or overeating	**Feb.:** food market, Harvard chemistry, citizen plainly, Commerce Secretary Wilbur, White House asks, scientists investigate, etc. **Mar.:** obesity, anemia, Iran temporarily releases, CDC issued warning, blood pressure, Obama holdover call fly, etc. **Apr.:** White House, force, Crozier, roosevelt, Peter Navarro, confirmed cases, etc.
PHQ5: Feeling bad about yourself	**Feb.:** worst treating, accelerate return jobs, tendency, investigating suspected cases, unwanted rolls, mistakenly released, vaccine, predicted kill, etc. **Mar.:** testing January aid, executive order medical, VP secazar, risking, embarrassment ugly, unnecessarily injured, etc. **Apr.:** invisible, house press, gross, insidious, irresponsible, shame, trump, worst, obvious consequences, etc.
PHQ6: Trouble concentrating on things	**Feb.:** dangerous pathogens, distracted, ignorant attacks, funding, camps, travel advisor, let alone watching, etc. **Mar.:** dogs, Fox news cloth, institute allergy, hands soap water, self-quarantined, Christ redeemer, valves, etc. **Apr.:** Theodore Roosevelt, confused, Dalglish, economy shrinks, U.S. commerce, etc.
PHQ7: Moving or speaking so slowly that other people could have noticed	**Feb.:** panic, Santa Clara, furious, wall street journal reports, pencedemic bus, dead birds, Tencent accidentally, unhinged disease control, etc. **Mar.:** Theodore, federal reserve, panic buy, councilwoman, anxiety, USS Theodore, frantic, avian swine, etc. **Apr.:** chief medical officer, social distancing, NHS lives, rallies jan, CDC issued warning, enrollment, Ron Desantis, etc.
PHQ8: Thoughts that you would be better off dead	**Feb.:** death people, China death, death toll rises, cut, China deadly outbreak, Hubei, lunar year, laboratories linked, first death, etc. **Mar.:** Washington state, dead, prevent, causing, worse, kill, death camps, increasing, etc. **Apr.:** death, patient, living expenses, abused, uninsured, treatment, death camps, etc.

## Appendix A

**Table A1:** Notation table.

Notation	Description
pattern pool	A subset of the extraction patterns that tend to extract the seed words
candidate word pool	The candidate nouns extracted by pattern pool are placed in candidate word pool
TC	Total correlation, also called multi-information, it quantifies the redundancy or dependency among a set of n random variables.
$D_{KL}$	Kullback–Leibler divergence, also called relative entropy, is a measure of how probability distribution is different from a second, reference probability distribution [50].
$p(x_{G})$	Probability densities of $X_{G}$
I(X:Y)	The mutual information between two random variables
p(y|x)	y’s dependence on x can be written in terms of a linear number of parameters which are just the estimate marginals
δ	The Kronecker delta, a function of two variables. The function is 1 if the variables are equal, and 0 otherwise.
$Z_{j}\left(x\right)$	A constant used to ensure the normalization of p(y|x) for each x. It can be calculated by summing |y|=k, an initial parameter.
